# Supplementary material for: Influence of Nutritional Ketosis Achieved through Various Methods on Plasma Concentrations of Brain Derived Neurotropic Factor
Source: Brain Sci. 2022 Aug 27;12(9):1143. doi: 10.3390/brainsci12091143 (PMC9496887; doi:10.3390/brainsci12091143)
Supplement: Supplementary file 1 [file brainsci-12-01143-s001.zip › brainsci-1833542-supplementary.pdf]

Supplementary Table S1 | Post-Hoc Comparisons for Significant (p < 0.05) RM ANOVA Effects

Study 1 | Main Effect of Time Post-hoc Comparisons

Bonferroni's multiple comparisons test	Predicted (LS) mean diff.	95.00% CI of diff.	Significant Effect?	Summary	Adjusted P Value
BL vs. IP	-127	-156.0 to -97.90	Yes	***	<.001
BL vs. IP30	-158	-186.7 to -129.4	Yes	***	<.001
IP vs. IP30	-31.08	-60.14 to -2.017	Yes	*	0.032

Study 1 | Main Effect of Condition Post-hoc Comparisons

Bonferroni's multiple comparisons test	Predicted (LS) mean diff.	95.00% CI of diff.	Significant Effect?	Summary	Adjusted P Value
KN+KS vs. KA+KS	0.9462	-51.79 to 53.68	No	ns	>.999
KN+KS vs. KN+WTR	36.66	-15.83 to 89.16	No	ns	0.361
KN+KS vs. KA+WTR	56.02	3.285 to 108.8	Yes	*	0.032
KA+KS vs. KN+WTR	35.72	-17.02 to 88.45	No	ns	0.408
KA+KS vs. KA+WTR	55.08	2.098 to 108.1	Yes	*	0.037
KN+WTR vs. KA+WTR	19.36	-33.38 to 72.10	No	ns	>.999

Study 1 | Interaction Post-hoc Comparisons

Bonferroni's multiple comparisons test	Predicted (LS) mean diff.	95.00% CI of diff.	Significant Effect?	Summary	Adjusted P Value
Baseline (BL)					
KN+KS vs. KA+KS	64.23	-8.102 to 136.6	No	ns	0.113
KN+KS vs. KN+WTR	86.22	13.89 to 158.5	Yes	*	0.011
KN+KS vs. KA+WTR	88.02	15.69 to 160.3	Yes	**	0.009
KA+KS vs. KN+WTR	21.99	-50.34 to 94.32	No	ns	>.999
KA+KS vs. KA+WTR	23.79	-48.54 to 96.12	No	ns	>.999
KN+WTR vs. KA+WTR	1.801	-70.53 to 74.13	No	ns	>.999
Immediately Post Exercise (IP)					
KN+KS vs. KA+KS	-74.5	-148.3 to -0.6942	Yes	*	0.047
KN+KS vs. KN+WTR	-75.3	-147.6 to -2.974	Yes	*	0.036
KN+KS vs. KA+WTR	-3.366	-77.17 to 70.44	No	ns	>.999
KA+KS vs. KN+WTR	-0.8067	-74.61 to 73.00	No	ns	>.999
KA+KS vs. KA+WTR	71.13	-4.115 to 146.4	No	ns	0.075
KN+WTR vs. KA+WTR	71.94	-1.865 to 145.7	No	ns	0.06
Immediately 30-min Post Exercise (IP30)					
KN+KS vs. KA+KS	13.11	-59.22 to 85.44	No	ns	>.999
KN+KS vs. KN+WTR	99.08	26.75 to 171.4	Yes	**	0.002
KN+KS vs. KA+WTR	83.42	11.09 to 155.7	Yes	*	0.015
KA+KS vs. KN+WTR	85.97	13.64 to 158.3	Yes	*	0.011
KA+KS vs. KA+WTR	70.31	-2.020 to 142.6	No	ns	0.062
KN+WTR vs. KA+WTR	-15.66	-87.99 to 56.67	No	ns	>.999

Study 3 | Main Effect of Time Post-hoc Comparisons

Bonferroni's multiple comparisons test	Predicted (LS) mean diff.	95.00% CI of diff.	Significant Effect?	Summary	Adjusted P Value
WK0 vs. WK2	120.5	67.03 to 174.1	Yes	***	**<.001**
WK0 vs. WK4	169.4	115.9 to 222.9	Yes	***	**<.001**
WK0 vs. WK6	246.8	193.2 to 300.3	Yes	***	**<.001**
WK2 vs. WK4	48.82	-4.693 to 102.3	No	ns	0.095
WK2 vs. WK6	126.2	72.70 to 179.7	Yes	***	**<.001**
WK4 vs. WK6	77.39	23.88 to 130.9	Yes	**	**0.001**

S1a

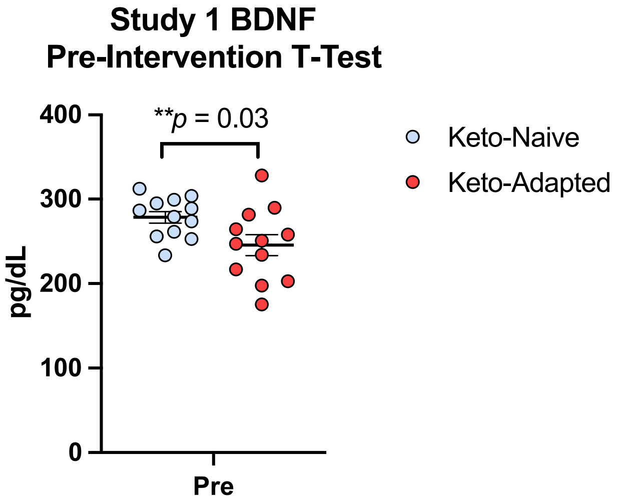

Supplementary Figure S1 | Study 1 Pre-Intervention T-Test

Pre-intervention measurements of BDNF were averaged between trials. A two-tailed T-Test was run between the two groups finding that the Keto-Naïve group had a higher Pre-intervention measurement of BDNF.

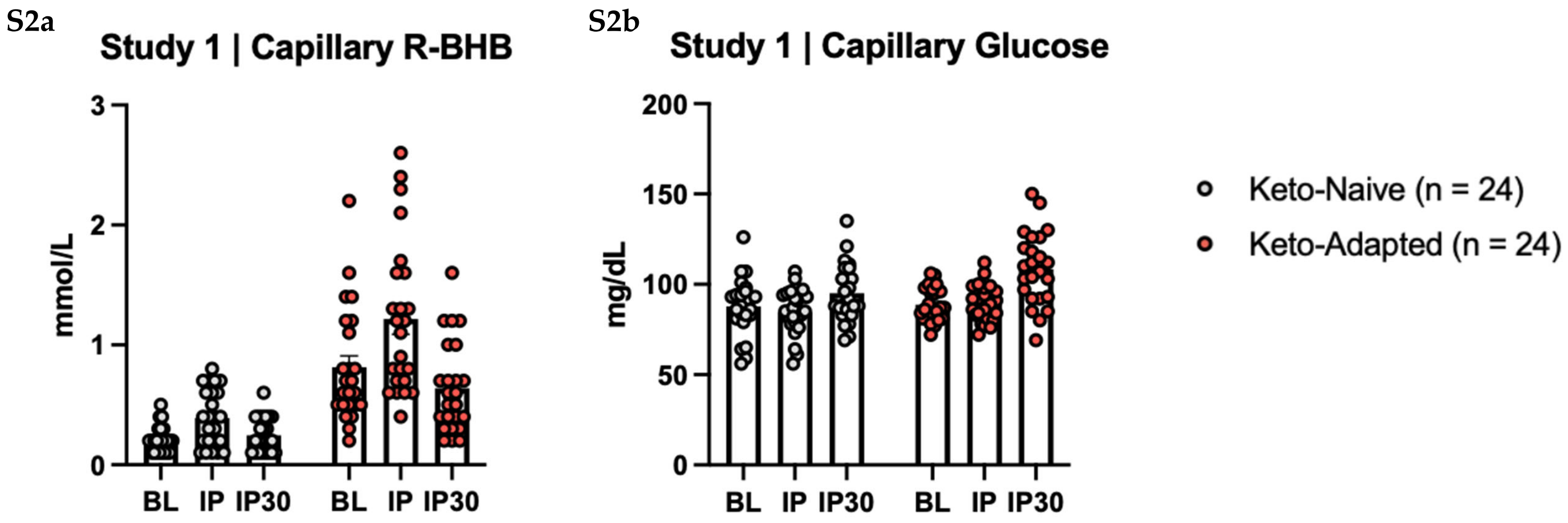

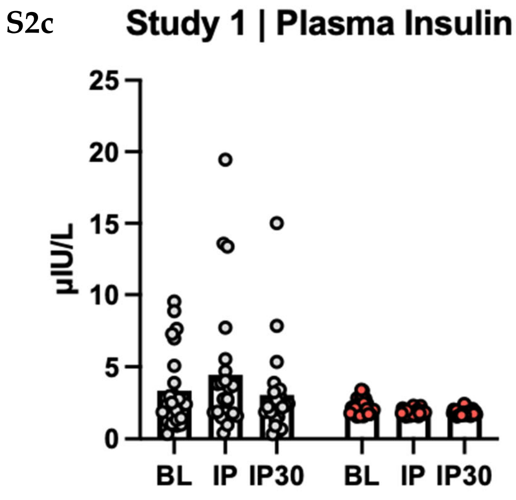

Supplementary Figure S2 | Study 1 Metabolic Marker Variance

Pre-intervention variations of Capillary R-BHB (Figure S2a), Capillary Glucose (Figure S2b), and Plasma Insulin (Figure S2c). Baseline measurements for each baseline trial were included.

S3a

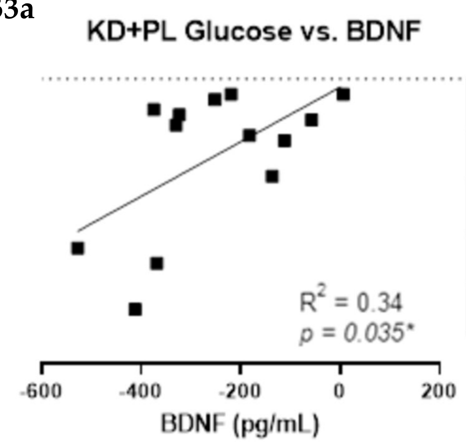

S3b

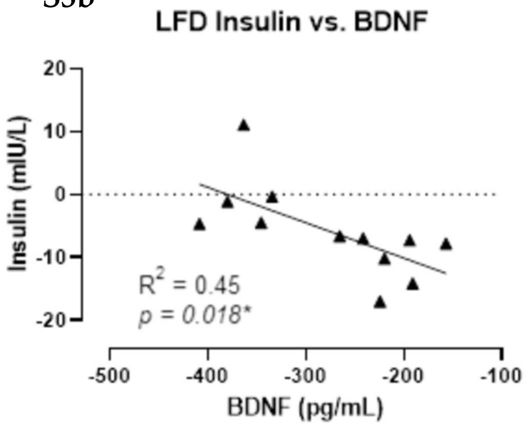

Supplemental Figure S3| Significant Correlations in Study 3

Significant correlations were found in Study 3. A positive correlation in the KD+PL group between BDNF and glucose concentration was detected ***(Figure S3a)****, while a negative correlation was found in the LFD group with plasma level insulin concentration and BDNF* ***(Figure S3b)***

Supplementary Table S2 | BDNF Pre- Post Change Correlations

	Experiment									Overall		
	Study 1				Study 2		Study 3			Study 1	Study 2	Study 3
	KA+KS	KA+WT	KN+KS	KN+WT	KD	MD	KD+KS	KD+PL	LFD			
BHB	0.24	0.35	0.19	-0.44	0.34		-0.54	-0.35	0.32	-0.02	0.34	-0.14
GLU	-0.27	-0.08	0.05	-0.28	-0.06	-0.35	-0.01	**0.59**	0.12	-0.02	-0.18	0.23
Insulin	0.22	0.10	-0.28	-0.31	-0.04	-0.35	0.40	0.17	**-0.67**	-0.09	-0.23	0.07
BW							-0.42	0.29	-0.32			-0.16
BF%							-0.24	0.43	0.15			0.16

KA, keto-adapted; KN, keto-naïve; KS, ketone salt; WT, water.
KD, ketogenic diet; PL, placebo; LFD, low-fat diet.
MD, mixed diet.

Data presented as r-value.
Values in **bold** face denote statistical significance ($p < 0.05$).
